# SAR1B GTPase is necessary to protect intestinal cells from disorders of lipid homeostasis, oxidative stress, and inflammation[S]

**DOI:** 10.1194/jlr.RA119000119

**Published:** 2019-08-13

**Authors:** Alain Sané, Lena Ahmarani, Edgard Delvin, Nikolas Auclair, Schohraya Spahis, Emile Levy

**Affiliations:** Research Centre,* CHU-Sainte-Justine, Université de Montréal, Montreal, Quebec, Canada; Departments of Pharmacology,† Université de Montréal, Montreal, Quebec, Canada; Nutrition,§ Université de Montréal, Montreal, Quebec, Canada

**Keywords:** gene silencing, SAR1 paralogues, triglyceride-rich lipoproteins, metabolism, mitochondria, intestine, transcription factors

## Abstract

Genetic defects in SAR1B GTPase inhibit chylomicron (CM) trafficking to the Golgi and result in a huge intraenterocyte lipid accumulation with a failure to release CMs and liposoluble vitamins into the blood circulation. The central aim of this study is to test the hypothesis that *SAR1B* deletion (*SAR1B^−/−^*) disturbs enterocyte lipid homeostasis (e.g., FA β-oxidation and lipogenesis) while promoting oxidative stress and inflammation. Another issue is to compare the impact of *SAR1B^−/−^* to that of its paralogue *SAR1A^−/−^* and combined *SAR1A^−/−^*/*B^−/−^*. To address these critical issues, we have generated Caco-2/15 cells with a knockout of *SAR1A*, *SAR1B*, or *SAR1A/B* genes. *SAR1B^−/−^* results in lipid homeostasis disruption, reflected by enhanced mitochondrial FA β-oxidation and diminished lipogenesis in intestinal absorptive cells via the implication of PPARα and PGC1α transcription factors. Additionally, *SAR1B^−/−^*cells, which mimicked enterocytes of CM retention disease, spontaneously disclosed inflammatory and oxidative characteristics via the implication of NF-κB and NRF2. In most conditions, *SAR1A^−/−^* cells showed a similar trend, albeit less dramatic, but synergetic effects were observed with the combined defects of the two *SAR1* paralogues. In conclusion, *SAR1B* and its paralogue are needed not only for CM trafficking but also for lipid homeostasis, prooxidant/antioxidant balance, and protection against inflammatory processes.

The small intestine is the essential site for the transport of alimentary fat in the form of lipoproteins. After the digestive phase, the lipolytic products in the lumen are absorbed by enterocytes that possess the unique ability to elaborate chylomicrons (CMs), the largest triglyceride (TG)-rich lipoprotein and dietary lipid vehicle (1, 2). CM assembly within epithelial absorptive cells is a multistep pathway that includes the translocation of lipolytic products from the apical membrane to the ER by cytosolic fatty acid-binding proteins (FABPs), lipid esterification, synthesis, and the posttranslational modification of different apos, especially apoB-48, as well as the packaging of lipid and apo components into pre-CM particles (3–5). It is only under these specific conditions that CM particles move huge amounts of dietary fat into the blood circulation via the lymphatic system.

Accidents of nature reveal the intracellular roles of key proteins in CM assembly and secretion (6). For example, deciphering chylomicron retention disease (CRD) afforded new insight into the crucial functions of SAR1B GTPase, the defects of which lead to severe fat malabsorption, hypocholesterolemia, extensive steatorrhea, and significant failure to thrive in children (7, 8) with developmental abnormalities in various organs (9). It is now well established that mutations in *SAR1B* (*SARA2*) gene encoding the SAR1B GTPase protein prevent the coat protein complex II (COPII) from producing mature CM-contained vesicles endowed with the ability to bud from the ER and reach the Golgi apparatus (6, 10, 11). Therefore, the genetic defects in *SAR1B* inhibit the step of CM trafficking to the Golgi and result in a huge accumulation of intraenterocyte TG with a failure to release CM and liposoluble vitamins into the blood circulation (2, 12).

Recently, we have shown that the total silencing of *SAR1B *(*SAR1B^−/−^*) lessens but does not extinguish the output of TG-rich lipoproteins (13) in intestinal cells, as is the case for patients with CRD (6, 10, 14). It is only through the combined depletion of *SAR1B* and *SAR1A* that CM delivery is fully eliminated. Additionally, *SAR1B^−/−^* modulates the protein expression of intestinal FABP, hepatic FABP, and the microsomal TG transfer protein while decreasing ATP-binding cassette transporter A1, thereby affecting enterocyte cholesterol efflux and high-density lipoprotein generation (13). However, how the absence of CM mobilization and secretion, due to *SAR1B* defects, influences lipid homeostasis in enterocytes remains an intriguing issue. Specifically, we have no information as to the impact of overwhelming lipid accumulation on intracellular FA β-oxidation on the one hand and on lipogenesis on the other. Furthermore, another question posed in the current study is the extent of intracellular oxidative stress (OxS) and inflammation provoked by *SAR1B* defect-derived steatosis. Finally, the contribution of or the complementarity between the distinct *SAR1* paralogues, *SAR1A* and *SAR1B*, in the enterocyte lipid homeostasis remains an open question.

Therefore, the major aim of this study is to test the hypothesis that *SAR1^−/−^*-mediated lipid accumulation influences intracellular lipid metabolic pathways (β-oxidation and lipogenesis) and affects OxS and inflammation via the modulation of major transcription factors. To address these critical issues, we have generated Caco-2/15 cells with a disruption of *SAR1A *(*SAR1A^−/−^*), *SAR1B^−/−^*, or combined *SAR1A^−/−^*/*B^−/−^* genes. In these engineered cells, we examined mitochondrial oxidative flux with a focus on critical factors (carnitine palmitoyltransferase 1 and acyl CoA dehydrogenase), lipogenesis with a particular emphasis on regulatory enzymes (acetyl CoA carboxylase and fatty acid synthase), lipophagy by stressing perilipin 2 (PLIN2) protein expression, and mechanisms by punctuating the mass of transcription factors.

## MATERIALS AND METHODS

### Generation of *SAR1* KO cells

This procedure was carried out as described previously (13). In short, zinc finger nuclease constructs against human *SAR1B* (CKOZFN18849) were transduced in proliferating Caco-2/15 cells according to the manufacturer’s guidelines to generate an *SAR1B^−/−^* clone. *SAR1A* clustered regularly interspaced short palindromic repeats (CRISPRs) and CRISPR-associated protein 9 (Cas9) KO constructs (sc-404190; Santa Cruz Biotechnology) were also used in parallel to create *SAR1A^−/−^* cells. SAR1 double KO (*SAR1A^−/−^*/*B^−/−^*) was then established by transfecting *SAR1B^−/−^* cells with the previous *SAR1A* CRISPR and Cas9 KO constructs. Cell viability was assessed via trypan blue staining. The clones for single (*SAR1B^−/−^*or* SAR1A^−/−^*) and double (*SAR1A^−/−^*/*B^−/−^*) KOs were then assayed for ablation through quantitative RT-PCR (qRT-PCR) and immunoblotting. Nontransfected cells were used as controls.

### Cell culture

Caco-2/15 cells were cultured as described previously (15). Briefly, they were grown at 37°C with 5% CO_2_ in MEM containing 1% penicillin/streptomycin, 1% MEM nonessential amino acids, and 5% decomplemented FBS. For experiments, cells were plated at a density of 1 × 10^6^ cells/well on 6-well polycarbonate plates (Costar). Prior to any experiment, cells were cultured for 14 days to fully differentiate into a monolayer with typical properties of absorptive enterocytes.

### Integrity of SAR1 KO cells

Cell integrity was assessed by determining villin protein expression as a hallmark of cell differentiation (16). Transepithelial electric resistance was also measured to validate the monolayer regularity and the tight junction dynamics reliability (17).

### Lipid synthesis

Caco-2/15 cells were preincubated overnight in serum-free medium supplemented with 1 μmol unlabeled oleic acid. The cells were then washed with PBS and incubated for 4 h in the presence of 5 μCi [^14^C]acetate. After the incubation period, the cells were washed and scraped with a scraper in a PBS solution containing antiproteases (1 mM phenylmethylsulfonyl fluoride, 1 mM pepstatin, and 1 mM Trasylol). Cellular [^14^C]labeled FAs were separated by TLC using the solvent mixture of hexane, ether, and acetic acid (80:20:3; v/v/v). The area corresponding to FFAs was scratched off the TLC plates, and the silica powder was placed in a scintillation vial with Ecolite counting fluid cocktail (MP Biomedicals). Radioactivity was then measured by scintillation counting with a HIDEX 300 SL automatic liquid scintillation counter (Southern Scientific). Cell protein was quantified using the Bradford method, and results were expressed as disintegrations per minute per milligram of cell protein.

### FA β-oxidation measurement

Differentiated Caco-2/15 cells were preincubated overnight in serum-free medium containing 1 μmol unlabeled oleic acid. The cells were then trypsinized and washed in warm PBS prior to resuspension in 2 ml serum-free medium containing 0.6 μCi/ml [^14^C(U)]palmitic acid conjugated with 10% FA-free BSA (pH 7.5). Cell suspensions were transferred into a mini-Erlenmeyer, in which a piece of hyamine hydroxide-wetted Whatman paper was placed to trap CO_2_ produced during the 2 h incubation at 37°C under shaking. After the incubation, 300 μl HCl (1N) was added to the cells, which were incubated for an additional 0.5 h at 37°C with shaking to stop the reaction. At the end, the pieces of Whatman paper were transferred to scintillation vials for radioactivity counting. Cell suspensions were centrifuged, and radioactivity in the supernatants was counted for the evaluation of acid-soluble metabolites.

### Mitochondrial imaging

Mitochondrial morphology was visualized using Mitotracker CMXRos (Red) solution (Invitrogen) according to the manufacturer’s instructions. Briefly, after an incubation of 12 days, Caco-2/15 cells were washed with warm PBS, and the culture medium was replaced with serum-free DMEM containing 50 nM MitoTrackerR probe (30 min at 37°C). Once again, Caco-2/15 cells were washed, and serum-free DMEM was added for viewing with an inverted, spinning-disk confocal microscope (Leica-DMi8) with a 63× oil objective and CMOS select camera at a laser emission of 560 nm. Using Visiview software, live-cell imaging and z-stacks were generated. The 3D cell intensities were measured from z-stacks with ImageJ software and z-projections of the average intensity.

### Lipid peroxidation monitoring

The amount of free malondialdehyde (MDA), a lipid peroxidation marker, produced in Caco-2/15 cells following treatment with palmitate was measured in cell extracts by HPLC with fluorescence detection. The protein-free supernatants were obtained after protein precipitation with a 0.44 M phosphoric acid solution. They were then reacted with an equivalent volume of 0.5% (w/v) thiobarbituric acid solution (Sigma-Aldrich) at 95°C for 1 h. After cooling to room temperature, the pink chromo gene [(TBA) 2-MDA] was extracted with 1-butanol and dried under a nitrogen stream at 37°C. The dry extract was finally dissolved in water before MDA determination with a reversed-phase HPLC method with a diode array detector set at 532 nm. In parallel, the antioxidant defense was evaluated in the same cell extracts by assessing the expression level of glutathione peroxidase 1.

### Western blot determination

Differentiated Caco-2/15 cells were incubated with 0.25 μmol palmitate for 2 h. Cells were then lysed in ice-cold buffer containing 20 mM Tris-HCl (pH 7.5), 150 mM NaCl, 1 mM Na_2_EDTA, 1 mM EGTA, 1% NP-40, 1% deoxycholate, 2.5 mM sodium pyrophosphate, 1 mM Na_2_VO_4_, 1 μg/ml leupeptin, and 1 mM PMSF. Aliquots of homogenates containing 40 μg total proteins were subjected to 7.5% or 12% SDS-PAGE and electroblotted onto nitrocellulose membranes. These membranes were then incubated overnight at 4°C with the specific primary antibodies of SAR1A/B (1/1,000; kindly provided by Dr Randy Schekman, University of California, Berkeley); villin (1/1,000; BD Transduction); TNF-α (1/1,000; Abcam); peroxisome proliferator-activated receptor γ coactivator 1α (PGC-1α) (1/1,000; Abcam); PPAR-α (1/1,000; Cayman Chemical); acyl-CoA dehydrogenase long chain (ACADL) (1/1,000; Thermo Fisher Scientific); carnitine palmitoyl transferase 1α (CPT-1α) (1/1,000; Cell Signaling Technology); sterol regulatory element-binding protein 1 (SREBP-1c) (1/1,000; Abcam); uncoupling protein 2 (UCP-2) (1/1,000; Novus Biologicals); NF-κB p65 subunit (1/5,000; Santa Cruz Biotechnology); nuclear factor erythroid-2-related factor 2 (NRF2) (1/1,000; Abcam); inhibitor κBα (I-κBα) (Cell Signaling Biotechnology); glutathione peroxidase 1 (1/1,000; Novus Biologicals); PLIN2 (1/250; Progen); and β-actin (1/250,000; Sigma-Aldrich) used as an internal control. After incubation with the relative secondary antibody mouse IgG-POD/rabbit IgG-POD (1/10,000; Roche Diagnostics), immune complexes were revealed using Clarity Max Western ECL substrate (Bio-Rad). Reactive bands were captured using a ChemiDoc MP Imaging System (Bio-Rad). All data are expressed as the ratio of target protein to β-actin in the same sample.

### RNA extraction and real-time qPCR

RNA was extracted from the samples using the PureLink RNA Mini Kit (Ambion). RNA concentration and purity were measured using a Biodrop Touch Duo spectrophotometer (Montreal Biotech Inc.). All RNA samples had an A260/A280 ratio of 1.95/2.05. After DNase treatment (Invitrogen), cDNA was generated from 1 μg aliquots of total RNA using SuperScript VILO Master Mix (Invitrogen). Real-time-qPCR was performed using the 7500 Fast Real-Time PCR System (Applied Biosystems). The thermal profile included an initial denaturation at 95°C for 30 s, followed by 40 cycles of denaturation at 95°C for 3 s and annealing and extension at 60°C for 30 s. Amplified genes were quantified by fluorescence using the PowerUp SYBR Green Master Mix (Applied Biosystems). Levels of expression of target-gene mRNAs were calculated by the 2^−ΔΔCT^ method (13).

### Statistical analysis

All values are expressed as the mean ± SEM of at least two independent experiments carried out in triplicates. Data were analyzed by one-way ANOVA using Prism version 6.0 (GraphPad Software). *P *< 0.05 was considered significant.

## RESULTS

### SAR1 deletion and Caco-2/15 cell integrity

**Fig. 1.**
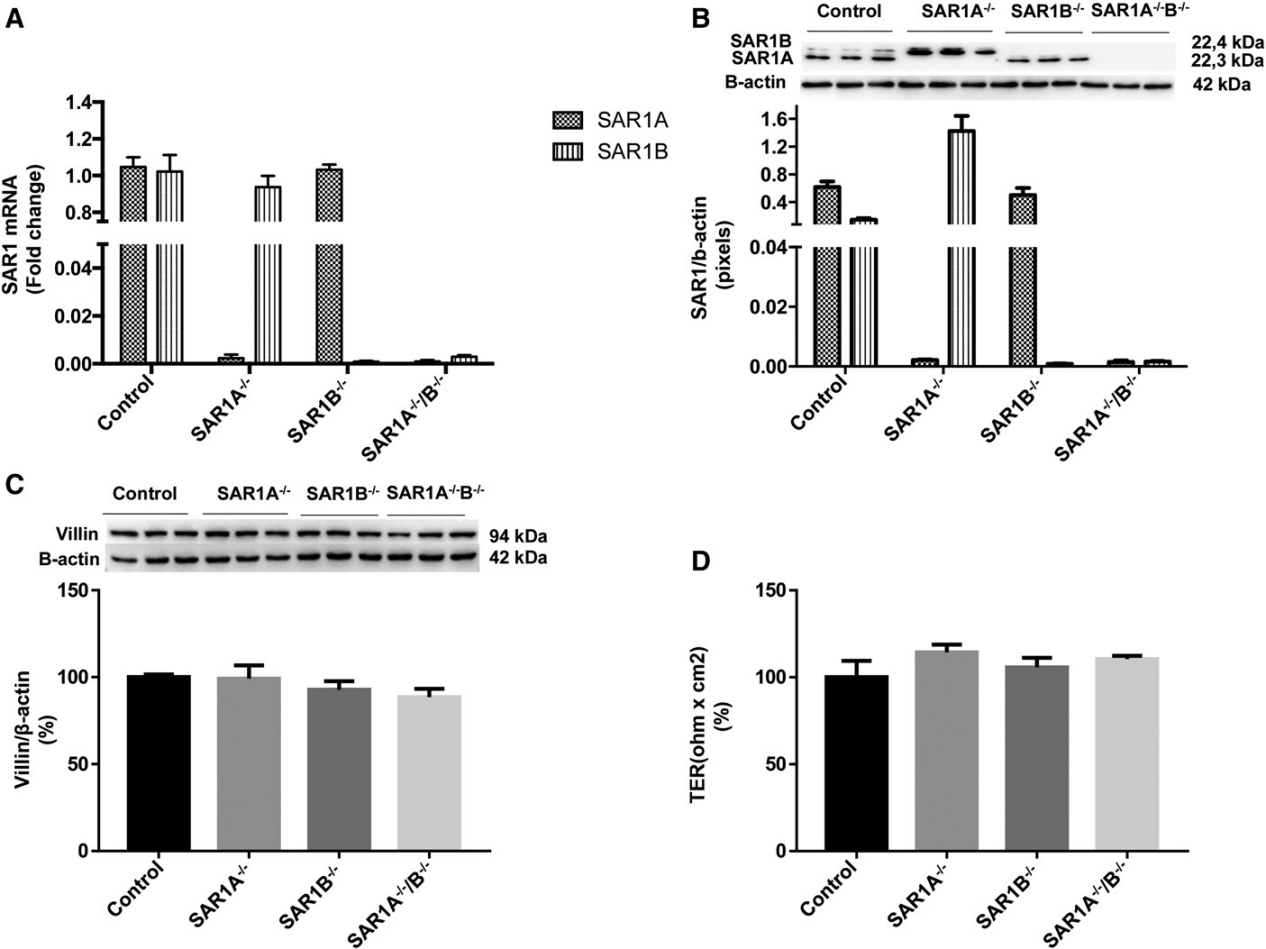
*SAR1* KO and integrity of genetically modified Caco-2/15 cells. Caco-2/15 cells were genetically engineered as described in the Materials and Methods section to produce cells with KO of *SAR1A, SAR1B*, or both paralogues. To validate the ablation efficiency, we determined the (A) gene and (B) protein expressions by qRT-PCR and Western blot, respectively. The genetic effect of manipulation on the cell monolayer integrity of Caco-2/15 cells was assessed by determining (C) villin protein expression as a marker of cell differentiation and (D) the transepithelial resistance (TER) as an index of cell permeability and tight junction constancy. Data are expressed as the mean ± SEM of two to three experiments in triplicates.

To dissect the impact of *SAR1* KO on FA metabolism, we generated Caco-2/15 cells with the ablation of *SAR1A*, *SAR1B*, or the combined two paralogues (*SAR1A/B*) as previously described (13). Gene (**Fig. 1A**) and protein (Fig. 1B) expression illustrate the total ablation of genes of interest in differentiated Caco-2/15 cells.

Moreover, we previously demonstrated that these genetic manipulations do not afflict cell integrity and viability or the differentiation process as measured by trypan blue exclusion and transepithelial resistance assays (13). We confirmed that genetic manipulation does not affect villin protein expression, a biomarker of cell differentiation, in *SAR1*-defective cells compared with controls (Fig. 1C). Transepithelial resistance measurements also showed no changes between controls and genetically modified Caco-2/15 cells (Fig. 1D). Therefore, the different cellular models are well suitable to analyze the role of *SAR1* genes in intestinal lipid homeostasis.

### Lipid peroxidation and antioxidant status in response to *SAR1* KO

**Fig. 2.**
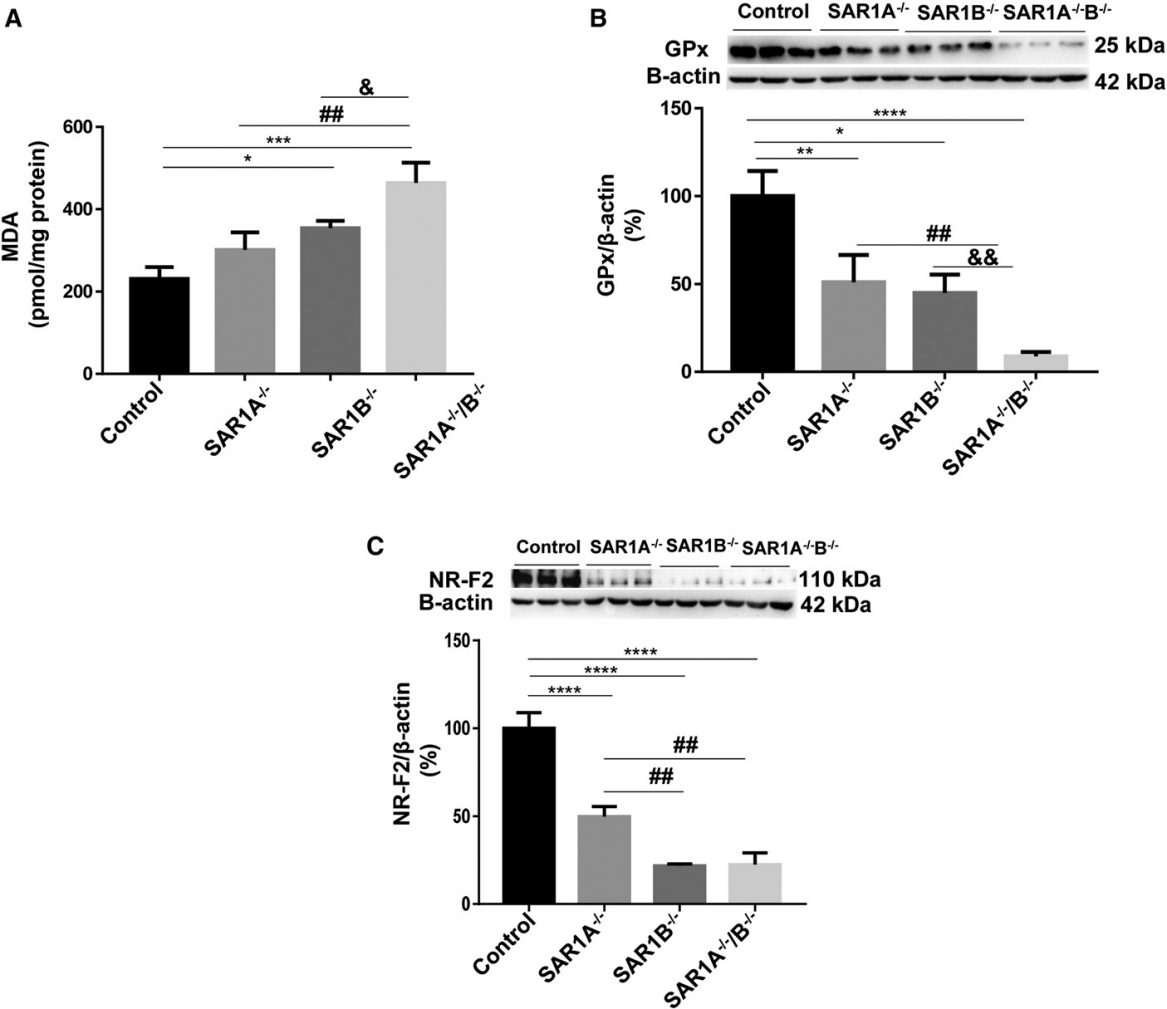
MDA levels and antioxidant response in *SAR1*-deleted cells. Differentiated Caco-2/15 cells were preincubated overnight with 1 μmol oleate and treated with 0.25 μmol palmitate for 2 h. Lipid peroxidation was evaluated by assessing (A) MDA content. Cells were also probed for (B) NRF2 and (C) GPx protein expression by Western blot. Data are expressed as the mean ± SEM of at least two experiments in triplicates. *P < 0.05, **P < 0.01, ***P < 0.001, and ****P < 0.0001 vs. controls; ^##^*P* < 0.01 vs. *SAR1A*^−/−^; and ^&^*P* < 0.05 and ^&&^*P* < 0.01 vs. *SAR1B^−/−^*.

A significant augmentation of MDA in *SAR1B^−/−^* cells and a consistent 2-fold increase in magnitude in *SAR1A^−/−^*/*B^−/−^* cells compared with controls were clearly observed, indicating lipid peroxidation (**Fig. 2A**). In view of these findings, we investigated the levels of expression of glutathione peroxidase (GPx), a protein whose role is to protect the organism from oxidative damage. GPx protein load was dramatically reduced in *SAR1^−/−^* cells, down to 9% in *SAR1A^−/−^*/*B^−/−^* cells compared with controls (Fig. 2B). We then turned to NRF2, a nuclear transcription factor that functions as the key controller of the redox homeostatic gene regulatory network. *SAR1^−/−^* cells showed a deep decrease in NRF2 protein expression, which particularly collapsed to 20% in *SAR1B^−/−^* and *SAR1A^−/−^*/*B^−/−^* (Fig. 2C). Concomitantly with the decline of NRF2, the observations support a breakdown of antioxidant defense in *SAR1^−/−^* intestinal cells.

### Modulation of inflammation by *SAR1* deletion

**Fig. 3.**
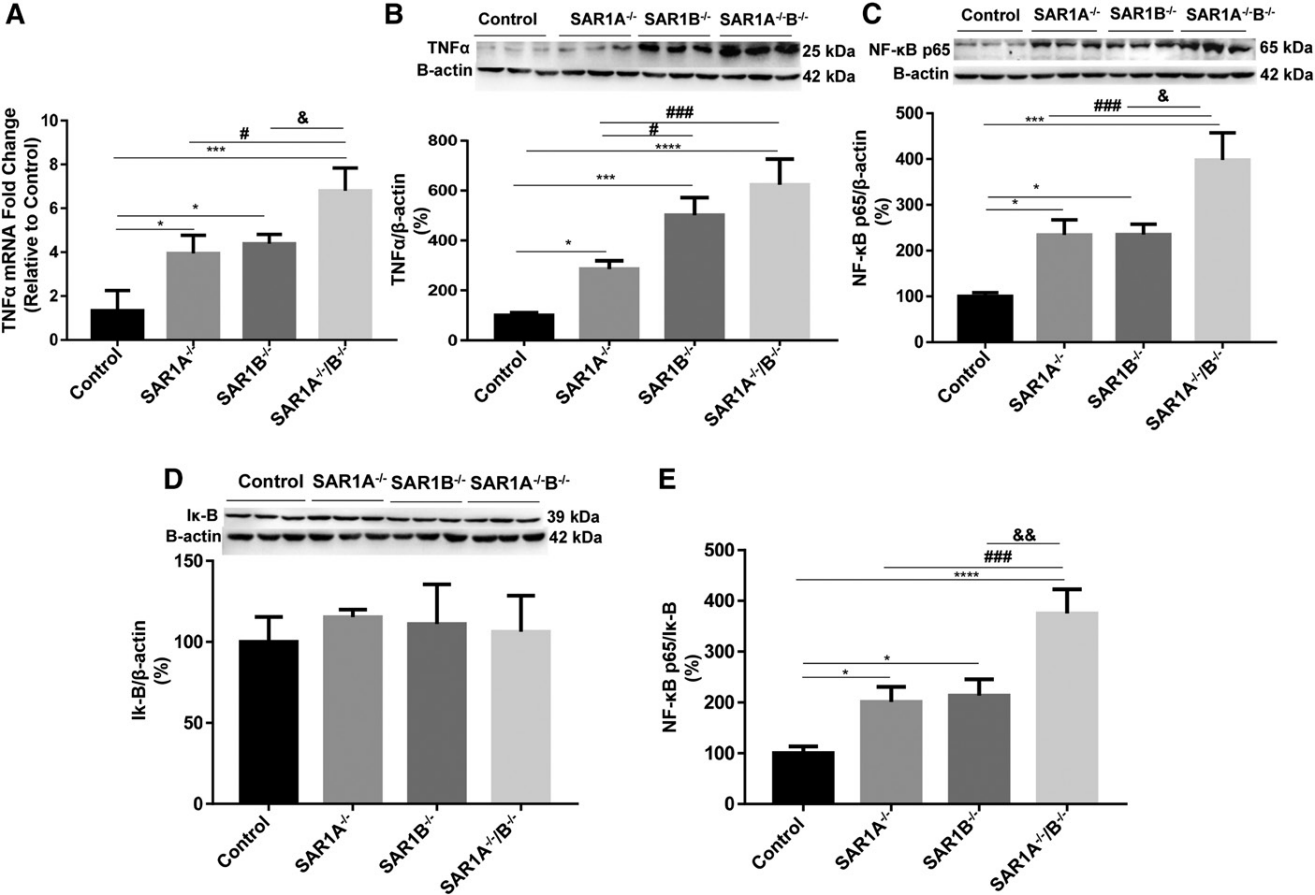
Impact of *SAR1* disruption on inflammatory markers. To rate the inflammation status, (A) gene expression, and (B) protein mass of TNF-α, (C) NF-κB p65 and (D) I-κB were assessed by qRT-PCR and Western blot, respectively. The (E) NF-κB/I-κB ratio was then calculated. Data are expressed as the mean ± SEM of two to three experiments in triplicates. *P < 0.05, ***P < 0.001, and ****P < 0.0001 vs. controls; ^#^*P* < 0.05 and ^###^*P* < 0.001 vs. *SAR1A^−/−^*; and ^&^*P* < 0.05 and ^&&^*P* < 0.01 vs. *SAR1B^−/−^*.

In view of *SAR1^−/−^*-derived lipid accumulation, it was necessary to evaluate inflammation. The TNF-α was significantly more expressed in *SAR1*-disrupted cells at the gene (**Fig. 3A**) and protein (Fig. 3B) levels, with more extended intensification in *SAR1A^−/−^*/*B^−/−^* cells. Because NF-κB is a key regulator of proinflammatory cytokines, we examined its potential activation in Caco-2/15 cells. Genetically modified cells showed a strong increase in NF-κB p65 protein expression in *SAR1A*^−/−^ and *SAR1B*^−/−^ that culminated with an almost 4-fold increase in *SAR1A*^−/−^/*B*^−/−^ cells (Fig. 3C) without any significant change in I-κB protein expression (Fig. 3D). The marked elevation of the NF-κB/I-κB ratio (Fig. 3E) confirmed the activation of NF-κB in *SAR1*^−/−^ cells.

### Impact of *SAR1* deletion on FA β-oxidation

**Fig. 4.**
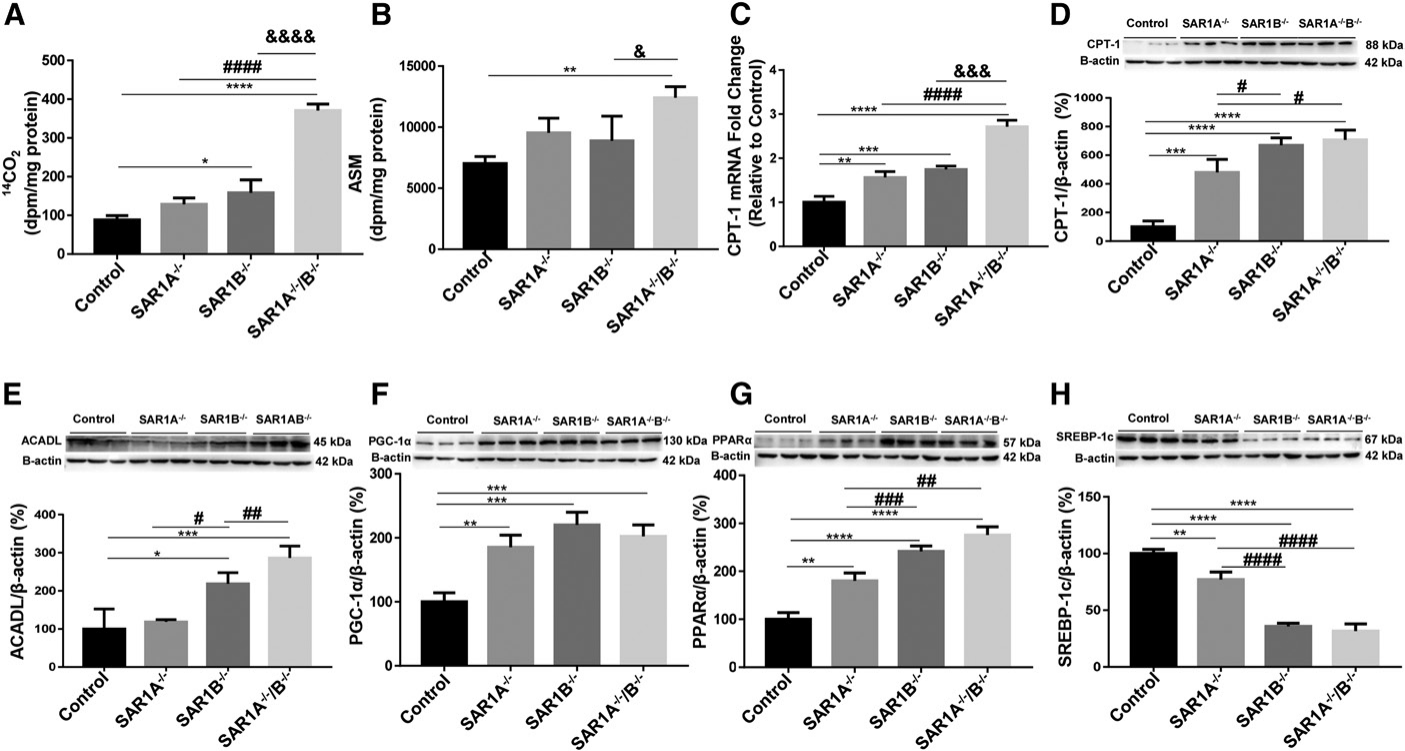
Effects of *SAR1* depletion on the production of ^14^CO_2_ and expression of major markers of FA β-oxidation. Cell suspensions of differentiated Caco-2/15 cells were incubated for 2 h with a reaction mixture containing 0.6 μCi/ml [^14^C(U)]palmitate. The amount of ^14^C in the (A) paper-disc CO_2_ fraction and (B) acid-soluble metabolite (ASM) fraction, respectively, was measured by scintillation counting. Furthermore, (C) mRNA and protein levels of (D) CPT-1α, (E) ACADL, (F) PGC-1α, (G) PPAR-α, and (H) SREBP-1c were determined by Western blot. Data are expressed as the mean ± SEM of at least two experiments in triplicates.*P < 0.05, **P < 0.01, ***P < 0.001, and ****P < 0.0001 vs. controls; ^#^*P* < 0.05, ^##^*P* < 0.01, ^###^*P* < 0.001, and ^####^*P* < 0.0001 vs. *SAR1A^−/−^*; ^&^*P* < 0.05, ^&&&^*P* < 0.001, and ^&&&&^*P* < 0.0001 vs. *SAR1B^−/−^*.

To test the impact of *SAR1*^−/−^ on mitochondrial oxidative flux, we incubated intestinal cells with [^14^C(U)]palmitate and, at the end of the incubation period, ^14^CO_2_ production was estimated. As shown in **Fig. 4A**, the degradation of the labeled palmitate was significantly increased in response to *SAR1B*^−/−^. While a similar trend of increased ^14^CO_2_ was noted in Caco-2/15 cells with *SAR1A^−/−^*, the results did not reach statistical significance. On the other hand, the double KO of *SAR1* paralogues led to a 4-fold increase in ^14^CO_2_ production compared with controls. Acid-soluble metabolites, which are the so-called intermediates of FA β-oxidation, were also augmented (75%), particularly in *SAR1A*^−/−^/*B*^−/−^ cells compared with controls (Fig. 4B). These results prompted us to appraise the mitochondrial expression of CPT-1α and ACADL because these proteins are rate-controlling enzymes of the FA β-oxidation pathway. A higher level of gene and protein expression characterized CPT-1α in response to *SAR1A*^−/−^, *SAR1B*^−/−^, and *SAR1A*^−/−^/*B*^−/−^ (Fig. 4C, D). Even if we did not succeed in estimating *ACADL* gene expression in Caco-2/15 cells, the assessment of ACADL protein expression disclosed elevated content in genetically modified Caco-2/15 cells relative to control cells with *SAR1B*^−/−^ and *SAR1A*^−/−^/*B*^−/−^, reaching 120% and 186%, respectively (Fig. 4E). On the whole, these findings sustain a stimulation of FA catabolic process through promoting the activities of control enzymes, while underscoring poor lipogenesis.

### A potent mechanism for *SAR1* deletion-mediated FA β-oxidation

We determined the protein mass of PPAR-α and PGC-1α because they represent two transcription factors necessary for the high-level expression of mitochondrial FA oxidation genes in conditions of *SAR1* deficiency. Western blot analysis showed a clear upregulation of PGC-1α (Fig. 4F) and PPAR-α (Fig. 4G) protein expression, with an increase of 80% to 175% and 85% to 120%, respectively, in *SAR1*-deficient cells. We also determined the protein expression of SREBP-1c, and the protein content was particularly low in *SAR1*-defective cells (Fig. 4H). As PGC-1α acts as a master regulator of mitochondrial function and biogenesis, it was interesting to examine mitochondrial content by assessing fluorescence after MitoTracker Red CMXRos staining. Examination by laser-scanning confocal microscope and analysis by ImageJ software (for processing images of individual 3D Caco-2/15 cells) allowed us to note only an increase in fluorescence intensity in *SAR1A*^−/−^/*B*^−/−^ (supplemental Fig. S1).

### FA synthesis and expression profiles of key enzymes of lipid synthesis

**Fig. 5.**
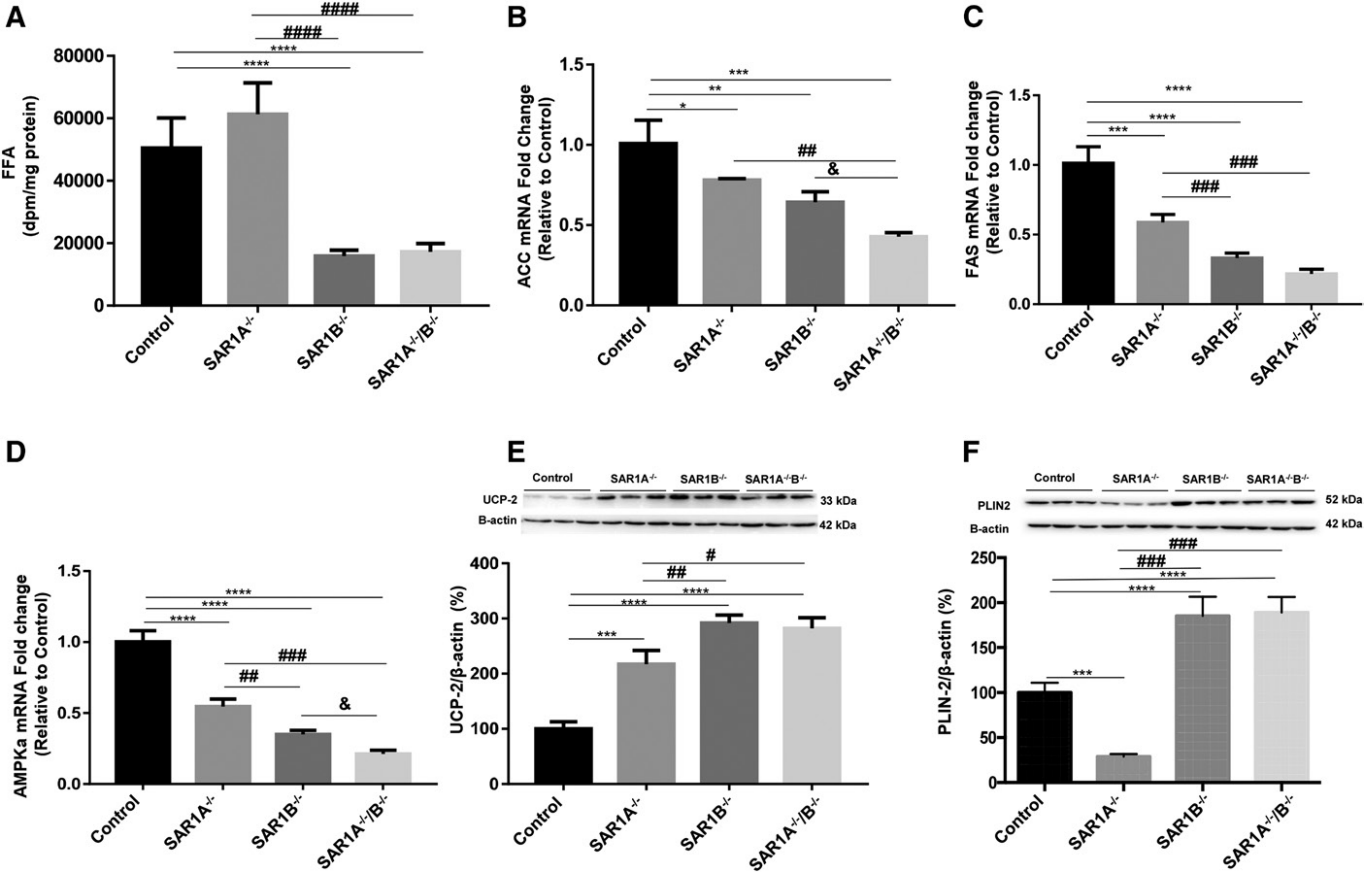
Impact of *SAR1* impairment on FA synthesis, lipogenesis biomarkers, and PLIN2, a regulatory factor for lipophagy. Differentiated Caco-2/15 cells were precultured overnight with FA substrate as described in the Materials and Methods section. Cellular [^14^C]FFAs were resolved by TLC. A: The area corresponding to FFAs was then counted for radioactivity and results expressed as disintegrations per minute per milligram of cell protein. Gene expression or protein content was evaluated for (B) ACC, (C) FAS, (D) AMPKα, (E) UCP-2, and (F) PLIN2. Data are presented as averages ± SEMs of at least two experiments in triplicates. *P < 0.05, **P < 0.01, ***P < 0.001, and ****P < 0.0001 vs. controls; ^#^*P* < 0.05, ^##^*P* < 0.01, ^###^*P* < 0.001, and ^####^*P* < 0.0001 vs. *SAR1A^−/−^*; and ^&^*P* < 0.05 vs. *SAR1B^−/−^*.

Cells were incubated with [^14^C]-acetate to estimate FFA synthesis in *SAR1*-depleted cells. As shown in **Fig. 5A**, de novo cellular FFA synthesis was radically decreased to 30% in *SAR1B^−/−^* and *SAR1A^−/−^*/*B^−/−^* cells without changes in *SAR1A^−/−^* cells. Accordingly, an important decline was noticed in acetyl-CoA carboxylase (ACC) and FAS mRNA (Fig. 5B, C). As AMP-activated protein kinase (AMPK) represents a central regulator of lipogenesis, we investigated its gene expression, which was lower in genetically altered cells to an extent of 80% in *SAR1A^−/−^*/*B^−/−^* cells (Fig. 5D). We also measured the protein expression of UCP-2 in genetically modified Caco-2/15 cells. As shown in Fig. 5E, the deletion of *SAR1* promoted UCP-2 protein expression 3-fold relative to controls.

### Lipophagy and PLIN-2

As lipophagy plays a significant role in the modulation of intracellular lipid metabolism and storage, we examined the status of PLIN-2, controlling how cells accumulate, mobilize, and utilize lipids. Western blot analysis revealed a decreased protein expression in *SAR1A*^−/−^ cells and an increased expression in *SAR1B*^−/−^ and *SAR1A*^−/−^/*B*^−/−^ cells (Fig. 5F).

## DISCUSSION

As CRD is highly characterized by impaired intestinal fat transport due to the inability to assemble and secrete CM, we have hypothesized that the deficiency of SAR1B leads to disturbed lipid homeostasis in enterocytes. Indeed, our experiments revealed a significant enhancement of FA β-oxidation in association with the upregulation of key mitochondrial enzymes such as CPT-1α and ACADL, driven by the induced expression of the transcription factors PPAR-α and PGC1-α. On the other hand, *SAR1* deletion lowered AMPKα mRNA expression, a central regulator of cellular energy homeostasis, as well as NRF2 protein mass, which serves as a major modulator of the cellular defense system against OxS. This caused a drop in GPx antioxidant protein and, consequently, an elevation of lipid peroxidation (MDA). Further, *SAR1* KO increased NF-κB and promoted inflammation (TNF-α). Finally, lipogenesis diminished in response to *SAR1* deletion, as reflected by the low levels of ACC, FAS, and SREBP-1c.

The critical issue before initiating the current study was to determine how the deletion of *SAR1B*, the gene responsible for CRD or Anderson’s disease (18, 19), affects intracellular lipid metabolism pathways. Our previous studies, using patients’ biopsies and genetically modified intestinal cells, clearly showed intracellular lipid retention and complete inability to secrete CMs (13, 18, 19). In fact, the molecular basis of this defect prevents the COPII to form a shell around the vesicles transporting CM cargo in the secretory pathway for their budding and fusion with Golgi, thereby impeding intestinal CM exocytosis (6). This is consistent with the longstanding severe deficiency of apoB-48 and circulating lipid-soluble vitamins even following a fat load (18, 19). Therefore, these earlier observations led us to the hypothesis that lipogenesis would be restricted while β-oxidation would be stimulated, and this assumption turned out to be true. Many factors may explain cellular homeostasis infringement in response to the *SAR1B* deletion, including the accumulation of lipids, abnormalities of protein transport, defects in nascent vesicle formation and membrane bending, poor recruitment of coat components, irregularities in the epithelial-mesenchymal transition, and alterations of cholesterol biogenesis (20, 21). Evidently, reasonable efforts must continue to understand how SAR1B, which regulates the formation and assembly of the ER-derived COPII vesicles and hence the trafficking of most cellular proteins, is involved in the control of lipid metabolic pathways.

We also assessed the repercussions of *SAR1B* deletion on diverse processes (e.g., OxS and inflammation) in order to better understand the metabolic events occurring in CRD patients. There is currently no genetic mouse model of intestinal CRD. The only way forward in this field is to undertake studies in the genetically modified cell line to introduce SAR1B abnormalities. These issues were tackled with Caco-2/15 cells, which spontaneously differentiate into polarized mature enterocytes under standard culture conditions. Importantly, the epithelial monolayer lends itself to the in vitro study of the human gut (particularly the jejunum segment), which optimally absorbs lipids, in view of its efficient intestinal transport processes and several morphological and functional features. In fact, this remarkable intestinal model is regarded as the most appropriate for the investigation of gut absorption and interactions, nutrition, toxicology food microbiology, bioavailability tests, and the screening of drug permeability in discovery programs. Multiple studies from our laboratory (22–39) and from other groups (40–44) have shown that Caco-2/15 monolayers are fully appropriate for the study of lipid/lipoprotein homeostasis. Furthermore, when seeded on porous filters (Transwell), Caco-2/15 cells permit access to both sides of the bipolar intestinal epithelium: apical and basolateral compartments, corresponding to intestinal lumen or serosal circulation, respectively. However, it would be important to confirm our findings by developing an *SAR1B^−/−^* mouse model.

Lipid accumulation is generally one of the most important manifestations of OxS. Various findings suggest that lipid accumulation is related to OxS in liver steatosis (45, 46). Another example is the ectopic lipid deposition in muscle, which has been shown to lead to OxS generation (47). Therefore, we have measured intestinal redox balance in response to *SAR1B* ablation. Our results show a spontaneous and significant elevation of lipid peroxidation along with vulnerable low GPx, a thiol-based enzyme that catalyzes the breakdown of damaging H_2_O_2_ and hydroperoxides to H_2_O and stable alcohols. The increased intracellular lipids resulting from *SAR1B* deletion may overwhelm the antioxidant defense and raise OxS in intestinal cells, which is consistent with the relationship between lipid accumulation and the generation of reactive oxygen species documented in earlier studies (48). Additionally, OxS may be attributable to amplified mitochondrial combustion of FAs because excessive FA oxidation leads to OxS and reduced antioxidant defenses (49). It is now well accepted that mitochondria constitute the foremost source of intracellular reactive oxygen species, as the electron transport consumes about 85% of the oxygen that the cell uses (50).

Moreover, we postulated that the *SAR1B*-defective intestinal cells spontaneously develop proinflammatory features in view of the elevated lipid content and OxS. Indeed, our results clearly demonstrate that *SAR1B* deficiency enhanced the production of TNF-α. It is likely that SAR1B^−/−^ exaggerated the production of cytokines through the transcription factor NF-κB, a powerful mechanism of inflammation. Interestingly, in our studies, UCP-2 was found to be highly upregulated, a strategy of survival in conditions of lipotoxicity and high OxS levels (51). The increased UCP-2 protein expression may be attributed to PPAR-α upregulation, as UCP-2 is a target gene of PPAR-α (52). Alternatively, UCP-2 may be activated by lipid content (53).

PPAR-α is intrinsically involved in mitochondrial fuel oxidation and biogenesis (54). As an imposing transcription factor, it regulates the expression of genes responsible for mitochondrial FA uptake and β-oxidation. In conditions of *SAR1B* deletion, the protein expression of PPAR-α had more than doubled, thus upregulating CPT-1α (critical for mitochondrial fatty acyl import) and ACADL (a rate-controlling enzyme of the β-oxidation pathway), which stimulated ^14^CO_2_ and acid-soluble metabolite production from [^14^C(U)]palmitate, indicative of an elevated rate of FA β-oxidation. The same trend was observed for *SAR1A* deletion and even more so when the two paralogues were deleted (*SAR1A^−/−^*/*B^−/−^*). As PGC-1α exerts pleiotropic effects (e.g., mitochondrial biogenesis and FA β-oxidation) by coactivating transcription factors such as PPAR-α (55), we assessed its expression by Western blot. Its protein mass was elevated, indicative of activation, which is in line with PPAR-α induction and FA β-oxidation enhancement. Furthermore, experiments with MitoTracker Red staining documented a significant increase in mitochondrial content in *SAR1A^−/−^*/*B^−/−^*, which suggests an increased mitochondrial mass and confirms PGC-1α findings. However, additional studies are necessary to evidence the stimulation of mitochondrial biogenesis.

SREBP-1c is the main modulator of FA synthesis by operating in the activation of lipogenic genes such as ACC and FAS (56). A stumpy expression characterized SREBP-1c, AMPKα, ACC, and FAS in *SAR1B^−/−^* cells, which is consistent with the decline in de novo FA synthesis. Overall, these findings reflect the drop in the lipogenic pathway. It is possible that PPAR-α is the master controller because various studies have shown that PPAR-α activation can suppress the activation of SREBP-1c (57–60).

As lipophagy plays a crucial role in lipid metabolism, it was important to determine its implication in the intestinal lipid accumulation caused by *SAR1B* deficiency. By definition, lipophagy is defined as the autophagic degradation of intracellular lipid droplets by the lysosomal acid lipase (61). It has recently been demonstrated that PLIN2 is a critical regulatory factor for lipophagy (62). For example, *PLIN2^−/−^* mice are characterized by a marked reduction of hepatic TG content and a sustained protection against fatty liver development (63). At the mechanistic level, a shortfall of PLIN2, acting as a shield for the TG core of cytosolic lipid droplets, enables lipases to access and hydrolyze TGs (64). As PLIN2 is largely expressed in the intestine and represents one of the most abundant lipid droplet coat proteins, with a profusion closely associated with the level of intracellular lipids (65, 66), we determined its protein expression in genetically modified Caco-2/15 cells. Our data showed a significant PLIN2 increase in *SAR1B^−/−^* and *SAR1A^−/−^*/*B^−/−^* but with a decline in *SAR1A^−/−^*, suggesting different mechanisms for the two paralogues.

Although one may speculate that the high levels of intracellular lipids may have pathophysiological implications for the intestinal mucosa of CRD patients in terms of cellular turnover rate and damage, the exploration of cell viability and differentiation did not show any defects in genetically modified Caco-2/15 cells. However, before totally dismissing this eventuality, measurements should be performed either in biopsies of CRD patients or in the small intestine of animal models engineered in the near future.

In conclusion, *SAR1B* silencing results not only in a failure to absorb lipids and fat-soluble vitamins in the form of CMs but also in lipid homeostasis disruption, reflected by enhanced mitochondrial FA β-oxidation and diminished lipogenesis in intestinal absorptive cells. Additionally, *SAR1B^−/−^* cells, which mimicked enterocytes of CRD, spontaneously disclosed inflammatory and oxidative characteristics. In most conditions, the combined defect in *SAR1A *and* SAR1B* promoted the severity of the aforementioned disorders.

## Supplementary Material

Supplemental Data
