# Supplementary material for: SAR1B GTPase is necessary to protect intestinal cells from disorders of lipid homeostasis, oxidative stress, and inflammation
Source: J Lipid Res. 2019 Aug 13;60(10):1755–64. doi: 10.1194/jlr.RA119000119 (PMC6795079; doi:10.1194/jlr.RA119000119)
Supplement: Supplemental Data [file supp_60_10_1755__index.html]

SAR1B IS NECESSARY TO PROTECT INTESTINAL CELLS FROM DISORDERS OF LIPID HOMEOSTASIS, OXIDATIVE STRESS & INFLAMMATION — SAR1B GTPase deletion disturbs enterocyte lipid homeostasis — SAR1B GTPase is necessary to protect intestinal cells from disorders of lipid homeostasis, oxidative stress, and inflammation — Supplemental Data 

# SAR1B GTPase is necessary to protect intestinal cells from disorders of lipid homeostasis, oxidative stress, and inflammation

## Supplemental Data

- Supplementary information - Supplementary information
