# Supplementary material for: SAR1B GTPase is necessary to protect intestinal cells from disorders of lipid homeostasis, oxidative stress, and inflammation
Source: J Lipid Res. 2019 Aug 13;60(10):1755–64. doi: 10.1194/jlr.RA119000119 (PMC6795079; doi:10.1194/jlr.RA119000119)

SAR1B GTPASE IS NECESSARY TO PROTECT INTESTINAL CELLS FROM DISORDERS OF LIPID HOMEOSTASIS, OXIDATIVE STRESS AND INFLAMMATION

Alain Sane[1], Lena Ahmarani[1], Edgard Delvin[1], Nikolas Auclair[1,2], Schohraya Spahis[1,3] and Emile Levy[1,2,3*]

[1]Research Centre, CHU-Sainte-Justine and Departments of [2]Pharmacology and [3]Nutrition, Université de Montréal, Montreal, Quebec, Canada

Running title: *SAR1B* deletion disturbs enterocyte lipid homeostasis

Corresponding author: *Dr. Emile Levy
GI-Nutrition Unit
CHU Sainte-Justine
3175 Ste-Catherine Road #4.17.005
Montreal, Quebec, Canada, H3T 1C5
Tel.: (514) 345-7783
E-mail: emile.levy@recherche-ste-Justine.qc

Primers used for qRT-PCR

Gene	Forward Primer	Reverse Primer
SAR1A	TTGGGCTTTATGGACAGACC	TTGAGCACACTGCACATGAA
SAR1B	TGCAGGAAAAACAACATTGC	GGTCAGTTCTTCGGAAGTGG
TNFa	GACAAGCCTGTAGCCCATGT	TTATCTCTCAGCTCCACGCC
CPT1a	ATTATGCCATGGATCTGCTG	AGCGGAGCAGAGTGGAATC
ACC	ACTTCGAGCACGCCAGGTTCT	TTCCGCCCATCCGCTGACAA
AMPKa	GACAGCCGAGAAGCAGAAAC	AGGATGCCTGAAAAGCTTGA
FAS	ATGGAGGAGGTGGTGATT	TGGCTTCATAGGTGACTTC
GAPDH	AGAAGGCTGGGGCTCATT	GGGCCATCCACAGTCTTCT

Supplementary Figure S1. Mitochondrial morphology

The 3D-cell intensities were measured from z-stacks with image J Software and z-projections of the average intensity were calculated as described in Method section. Data are presented as averages ± SEM of 10 cells/section. *P<0.05 vs. Controls.

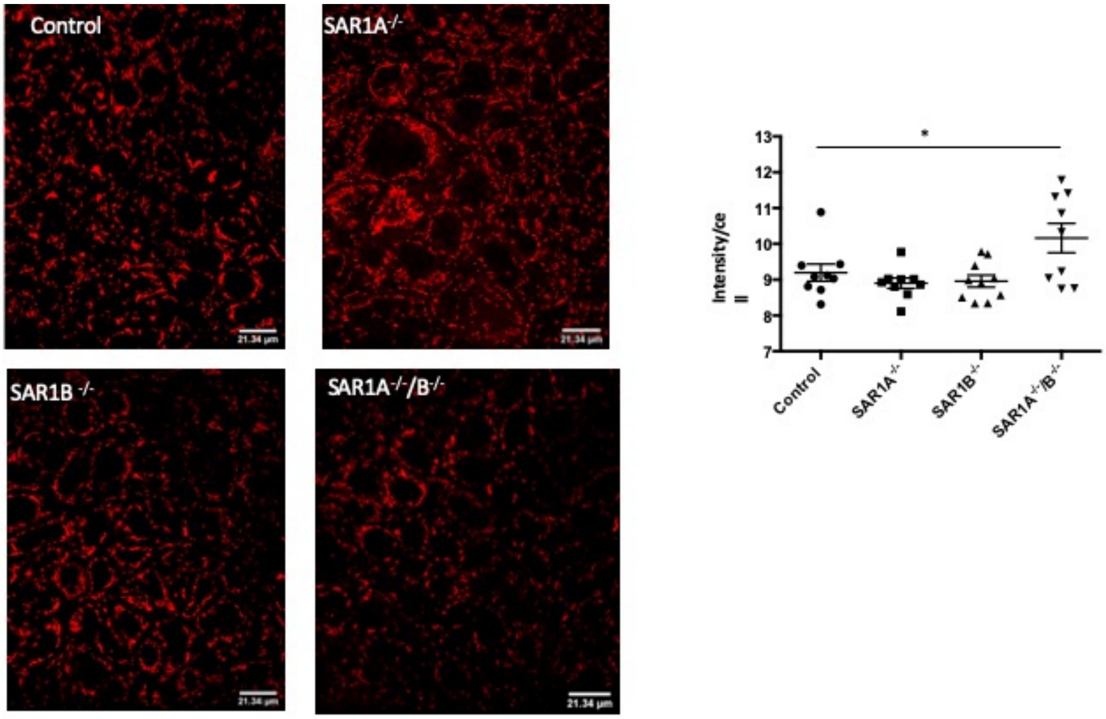

Supplement: Supplemental Data [file supp_RA119000119_152656_1_supp_368251_pvff8h.pdf]
